# Modeling of *LMNA*-Related Dilated Cardiomyopathy Using Human Induced Pluripotent Stem Cells

**DOI:** 10.3390/cells8060594

**Published:** 2019-06-15

**Authors:** Disheet Shah, Laura Virtanen, Chandra Prajapati, Mostafa Kiamehr, Josef Gullmets, Gun West, Joose Kreutzer, Mari Pekkanen-Mattila, Tiina Heliö, Pasi Kallio, Pekka Taimen, Katriina Aalto-Setälä

**Affiliations:** 1 BioMediTech, Faculty of Medicine and Health Technology; Tampere University, 33520 Tampere, Finland; chandra.prajapati@tuni.fi (C.P.); mostafa.kiamehr@tuni.fi (M.K.); Mari.Pekkanen-Mattila@tuni.fi (M.P.-M.); katriina.aalto-setala@tuni.fi (K.A.-S.); 2 Institute of Biomedicine, University of Turku, 20520 Turku, Finland; latevi@utu.fi (L.V.); josef.gullmets@helsinki.fi (J.G.); gun.west@utu.fi (G.W.); pepeta@utu.fi (P.T.); 3 Turku Doctoral Programme of Molecular Medicine, University of Turku, 20520 Turku, Finland; 4 Micro-and Nanosystems Research Group, BioMediTech, Faculty of Medicine and Health Technology, Tampere University, 33140 Tampere, Finland; joose.kreutzer@tuni.fi (J.K.); pasi.kallio@tuni.fi (P.K.); 5 Helsinki University Hospital, 00029 Helsinki, Finland; Tiina.Helio@hus.fi; 6 Department of Pathology, Turku University Hospital, 20520 Turku, Finland; 7 Medical School, University of Tampere, 33520 Tampere, Finland; 8 Heart Hospital, Tampere University Hospital, 33520 Tampere, Finland

**Keywords:** dilated cardiomyopathy, *LMNA*, Lamin A/C, induced pluripotent stem cell, hypoxia, microelectrode array and calcium imaging

## Abstract

Dilated cardiomyopathy (DCM) is one of the leading causes of heart failure and heart transplantation. A portion of familial DCM is due to mutations in the *LMNA* gene encoding the nuclear lamina proteins lamin A and C and without adequate treatment these patients have a poor prognosis. To get better insights into pathobiology behind this disease, we focused on modeling *LMNA*-related DCM using human induced pluripotent stem cell derived cardiomyocytes (hiPSC-CM). Primary skin fibroblasts from DCM patients carrying the most prevalent Finnish founder mutation (p.S143P) in *LMNA* were reprogrammed into hiPSCs and further differentiated into cardiomyocytes (CMs). The cellular structure, functionality as well as gene and protein expression were assessed in detail. While mutant hiPSC-CMs presented virtually normal sarcomere structure under normoxia, dramatic sarcomere damage and an increased sensitivity to cellular stress was observed after hypoxia. A detailed electrophysiological evaluation revealed bradyarrhythmia and increased occurrence of arrhythmias in mutant hiPSC-CMs on β-adrenergic stimulation. Mutant hiPSC-CMs also showed increased sensitivity to hypoxia on microelectrode array and altered Ca^2+^ dynamics. Taken together, p.S143P hiPSC-CM model mimics hallmarks of *LMNA*-related DCM and provides a useful tool to study the underlying cellular mechanisms of accelerated cardiac degeneration in this disease.

## 1. Introduction

Dilated cardiomyopathy (DCM) is a cardiac disorder characterized by weakening of the heart muscle due to a progressive loss of functional cardiomyocytes, dilation of cardiac ventricles, reduced cardiac output and arrhythmias [1]. Of all cases 25–30% are hereditary with an estimated incidence of 1:250–2500 [2,3]. Mutations in more than 30 different genes have been linked to the genetic form of DCM and the second most commonly mutated gene is *LMNA*, encoding nuclear lamin A and C proteins [3,4,5]. Lamins play an essential role in determining nuclear size, shape, stiffness and they have also been implicated in cell cycle progression, chromatin organization and DNA damage response [6]. A-type lamins, primarily lamin A and C, are derived through alternative splicing of the *LMNA* gene while B-type lamins (lamin B1 and B2) are encoded by two distinct genes, *LMNB1* and *LMNB2*, respectively. Over 500 mutations identified in the *LMNA* gene (http://www.umd.be/LMNA/) cause an exceptional variety of diseases commonly called laminopathies. In addition to DCM, these include e.g., muscular dystrophies, lipodystrophies, peripheral neuropathy and premature aging (progeria) [7], many of which also exhibit some features of cardiac disease.

A significant number of patients with *LMNA* mutations display complications only in the cardiovascular system and many remain undiagnosed [8]. Clinically, DCM patients and their family members carrying *LMNA* mutations should be identified for several reasons. First, the penetrance of the disease is nearly 100% among mutation carriers. Secondly, the cardiac dysfunction is almost always preceded by the conduction system disease, such as atrioventricular block, atrial fibrillation and sometimes potentially fatal ventricular arrhythmias or asystole [9]. Such patients with *LMNA* mutations are at a significantly higher risk of sudden death compared to other forms of DCM [10]. 92% of patients carrying *LMNA* gene mutations with either cardiac or neuromuscular phenotype were reported to present cardiac arrhythmias after the age of 30, 64% developed heart failure after the age of 50 and sudden death was the most common cause of death (46%) [11]. The current medical treatment includes general heart failure management with β-blockers and ACE inhibitors, but the existing therapy of DCM is not optimal [12,13]. Therefore, also intensively followed DCM patients with *LMNA* mutations have a poor prognosis and an intervention with a pacemaker or an implantable defibrillator, as well as cardiac transplantation, is occasionally needed [9]. 

The detailed mechanisms by which mutations in nuclear lamins cause DCM and cardiac dysfunction are still poorly understood but accumulating data from patients and animal models suggest that alterations in lamina structure initiate the onset of the disease by defective electrical signaling and molecular response to mechanical stress. Additionally, the mutations cause changes in chromatin organization and gene activity leading to altered gene expression and signaling and to progressive weakening of cardiac muscle; for review see [12,14].

Several mouse models have been established to study the pathophysiology of *LMNA*-related DCM [13,15,16,17]. Although important knowledge has been gained with the existing mouse models, generation of human induced pluripotent stem cells (hiPSC) provides an exceptional opportunity to make patient-specific cardiomyocytes (CMs) and study the underlying mechanism of DCM with human cardiac cells in vitro as reported in a hiPSC model for *LMNA*-related DCM model [18]. In accordance with the *Lmna*^H222P/H222P^ mouse model work [15], Siu et al. showed that MAPK inhibitors (U0126 and selumetinib) alleviate nuclear defects and apoptosis after electrical stimulation in hiPSC-CMs carrying the *LMNA*^R225X/WT^ mutation. In another study, application of Ataluren (PTC124) protected *LMNA*^R225X/WT^ hiPSC-CMs but not *LMNA*^Q354X/WT^ or *LMNA*^T518fs/WT^ hiPSC-CMs from nuclear abnormalities and apoptosis [19]. Ataluren also improved the excitation–contraction coupling and contractile functions in *LMNA*^R225X/WT^ hiPSC-CMs [19]. These studies emphasize the importance of personalized medicine for DCM treatment. In addition to DCM, hiPSC-CMs have been successfully used to model several other cardiac diseases e.g., hypertrophic cardiomyopathy [20], arrhythmogenic right ventricular cardiomyopathy [21] and long QT-syndrome [22]. Although iPSCs-CMs are a promising tool for disease modeling and drug discovery, the yield of mature cardiomyocytes, tumorigenic potential of the reprogrammed cells and the immune response of potential recipient require more research before any clinical applications [23].

In Finland, a heterozygous founder mutation p.S143P in *LMNA* is the most prevalent DCM-associated mutation with typical clinical phenotype [24]. We have previously shown that p.S143P mutation increases lamin A/C nucleoplasmicity, mobility and tendency to form intranuclear aggregates in patient fibroblasts and further activates unfolded protein response (UPR) [25]. In this follow-up work, hiPSC-CMs were generated from two individuals carrying the p.S143P *LMNA* mutation and the cellular structure, electrophysiological features and sensitivity to physiological stress (i.e., hypoxia) were compared to CMs from two healthy control individuals.

## 2. Materials and Methods

### 2.1. Patient Characteristics

Biopsies from two healthy controls and two patients carrying the p.S143P mutation in the *LMNA* [22,25] were used for this study. Healthy control cells were derived from a 55-year-old female (UTA.04602.WT) and from a 30-year-old male (UTA.11505.WT). Mutation carrier 1 (DCM1, UTA.12704.LMNA) is a 24-year-old male and mutation carrier 2 (DCM2, UTA.12619.LMNA) a 34-year-old female. DCM1 presented a high number of ventricular extrasystoles (9%) and one non-sustained ventricular-tachycardia (VT) period of 15 beats in ECG (electrocardiogram). His ejection fraction and serum brain natriuretic peptide levels were normal. DCM2 had a first-degree atrio-ventricular (AV) block and paroxysmal atrial flutter. Her ejection fraction was 41% at the lowest, but usually within the normal range. Both patients were on β-blocker therapy and had a family history of heart transplantation due to *LMNA* mutation. A signed informed consent was obtained from all the individuals participating in the study. The study was approved by the Ethics Committee of the Pirkanmaa Hospital District to establish, culture and differentiate hiPSC lines (R08070).

### 2.2. hiPSC Generation, Culture and Characterization

Two control and two DCM hiPSC lines were generated. Derivation of one control line (UTA.04602.WT) had been reprogrammed by lentiviral infection and characterized previously [22,26]. The second control UTA.11505.WT and two patient lines UTA.12704.LMNA and UTA.12619.LMNA were generated by sendai virus infection and all the lines were characterized similar to the control line UTA.04602.WT. Two control and two mutant cell lines were used throughout the study. However, due to lower differentiation efficiency of control2 line, the data from control1 and control2 was combined, unless otherwise indicated.

### 2.3. Cardiomyocyte Differentiation

hiPSCs were differentiated into cardiomyocytes as described earlier [27] using KO-DMEM (GIBCO, Invitrogen, Carlsbad, CA, USA) supplemented with CHIR99201 and IWP (inhibitor of WNT pathway) in B27 (GIBCO). This method yielded beating cardiac cultures within 8–10 days. All cardiac cells were maintained in KO-DMEM supplemented with 20% FBS and let to mature for at least 30 days before dissociation and use in experiments.

### 2.4. Genotyping

DNA sequencing was performed to confirm the presence of the p.S143P mutation in the patient-derived hiPSCs lines. Genomic DNA was extracted from hiPSC lines (Macherey Nagel DNA, RNA protein purification NucleoSpin Tissue XS kit, Düren, Germany). A PCR fragment of 776 bp around the *LMNA* mutation was amplified using polymerase chain reaction (PCR). The PCR was done using forward Primer *LMNA*_Fwd-GGCTCAGATCGAGAAGTGCTAGGGA, and reverse Primer *LMNA*_Rev-ATGACTCTAGGACAGGTGAATGGCTCTG at conditions 95 °C 2.30 min, then 30 cycles (95 °C, 0.30 min; 59 °C, 0.30 min; 72 °C, 0.50 min), a final extension at 72 °C 10 min and 4 °C cooling. The PCR product (776 bp) was electrophoresed on a 1% agarose gel and purified (NucleoSpin Gel and PCR Clean up Kit from Macherey-Nagel, Düren, Germany). Sequencing was carried out with the same forward primer. The product was then sent for sequencing at the Tampere facility of NGS and Sanger Sequencing.

In order to verify that the *LMNA* mutation is transcribed in the hiPSC-CMs, we carried out a qRT-PCR with mutation and wild type specific primers. One μg of RNA was converted to cDNA using a SensiFAST cDNA Synthesis kit (Bioline, London, UK). The amplification step was performed using a SensiFAST SYBR Lo-ROX qPCR kit (Bioline) according to the manufacturer’s protocol as follows: 3 min at 95 °C followed by 40 cycles of 5 s at 95 °C, 10 s at 60 °C and 15 s at 72 °C. Target genes were normalized to GAPDH expression and the values were calculated using the 2−ΔΔCt method. The primers used were for GAPDH 5′-TAAATTGAGCCCGCAGCCTCCC-3’ and 5′-ATGTGGCTCGGCTGGCGACG-3’; *LMNA* TOTAL 5’-GGGATGCCCGCAAGACCCTT-3’ and 5’-GGTATTGCGCGCTTTCAGCTCC-3’; *LMNA* WT 5’-GCTCTGCTGAACTCCAAGGAGG-3’ and 5’-GCCTCAAGCTTGGCCACCTG-3’; *LMNA* S143P 5’-GCTCTGCTGAACCCCAAGGAGG-3’ and 5’-GCCTCAAGCTTGGCCACCTG-3’.

### 2.5. Immunofluorescence and Confocal Microscopy

Dissociated CMs were fixed in 10% formalin for 10 min and permeabilized with 0.1% Triton X-100 for 10 min. For the ischemic stress (hypoxia and serum/glucose deprivation) induction, CMs were washed twice with 1× PBS and transferred in 1% O_2_ in a hypoxic workstation (Invivo2 400, Ruskinn Technology, Bridgend, UK) with oxygen replaced by 99.5% pure N_2_ (AGA, Espoo, Finland). Degassed serum and glucose deficient medium was changed inside the hypoxic workstation. After hypoxia (2–5 h) cells were washed twice with degassed 1× PBS and fixed inside the workstation.

The primary antibodies used were goat monoclonal Troponin T (cTnT, 1:2500, ab64623, Abcam, Cambridge, UK), mouse α-actinin (1:800, A7811, Sigma-Aldrich, Saint Louis, MO, USA), rabbit polyclonal lamin A (1:1000, 323-10, kindly provided by prof. Robert D. Goldman, Northwestern University, USA) and mouse monoclonal hypoxia-inducible factor 1-α (Hif-1α, 1:1000, clone-54, BD Biosciences, Franklin, NJ, USA). The secondary antibodies were donkey anti-rabbit IgG conjugated to Alexa Fluor 488, donkey anti-mouse IgG conjugated to Alexa Fluor 555 and goat anti-chicken IgG conjugated to Alexa Fluor 647 (all Molecular Probes, Eugene, OR, USA). ProLong Diamond Antifade Mountant with DAPI was used to visualize DNA (Thermo Fisher Scientific, Waltham, MA, USA).

The spinning disk confocal microscope used was a 3i Marianas with Yokogawa CSU-W1 scanning unit on an inverted Zeiss AxioObserver Z1 microscope, controlled by SlideBook 6 software (Intelligent Imaging Innovations GmbH, Göttingen, Germany). The objective used was 63×/1.4 oil. Images were acquired with ORCA Flash4 sCMOS camera (Hamamatsu Photonics, Hamamatsu, Japan) and analyzed with BioImageXD [28] and ImageJ Fiji software [29]. 20 to 30 nuclei were randomly selected and the mean area, circularity and fluorescence intensities of lamin A within the lamina (L) and nucleoplasmic (N) regions were quantified with ImageJ Fiji. The ratio of fluorescence between the lamina and nucleoplasma were calculated as follows:

$$intensity\;ratio=\frac{\left(N-B\right)}{\left(L-B\right)},$$

where B is the background. The sarcomere length and organization analysis was done with ImageJ plugin TTorg as previously descripted [30]. A two sample *t*-test was used to analyze the differences between control and DCM-CMs in nucleoplasmicity, sarcomere length and organization. *p* < 0.05 was considered statistically significant.

### 2.6. Transmission Electron Microscopy

Dissociated CMs were fixed with 5% glutaraldehyde in 0.16 M s-collidine buffer, pH 7.4, post fixed with 2% OsO_4_ containing 3% potassium ferrocyanide for 2 h, dehydrated with different ethanol concentrations (70%, 96%, 2 × 100%) and embedded with a 45359 Fluka Epoxy Embedding Medium kit. 70 nm sections were cut with an ultramicrotome and stained with 1% uranyl acetate and 0.3% lead citrate. The images were acquired with a JEOL JEM-1400 Plus TEM (Tokyo, Japan) equipped with a OSIS Quemesa 11 Mpix bottom-mounted digital camera operated at 80 kV acceleration voltage.

### 2.7. Immunoblotting

CMs were lysed in a M-PER mammalian protein extraction reagent (Thermo Fisher Scientific) complemented with 1× protease and 1× phosphatase inhibitors. Hypoxia-treated cells were lysed inside the hypoxia workstation. Protein concentration was measured using Pierce Coomassie Plus Protein Assay kit (Thermo Fisher Scientific). Cell lysates were mixed with 2× SDS-PAGE sample buffer and 15 μg of protein lysate was run on a 4–10% gradient gel (BioRad, Hercules, CA, USA). The primary antibodies used were mouse monoclonal anti-actin (1:1000, AC-40, Sigma-Aldrich), mouse monoclonal lamin A/C (LAC, 1:10,000, 5G4, kindly provided by Prof. Robert D. Goldman, Northwestern University, USA), mouse monoclonal heat shock protein 90 (Hsp90, 1:2000, ADI-SPA-830, Enzo Life Science, Farmingdale, NY, USA), mouse monoclonal heat shock protein 70 (Hsp70, 1:2000, ADI-SPA-810, Enzo Life Science), mouse monoclonal heat shock protein 60 (Hsp60, 1:1000, D85, Cell Signaling Technology, Danvers, MA, USA), rabbit monoclonal anti-peIF2α (1:1000, 119A11, Cell Signaling Technologies), rabbit monoclonal phospho-p44/42 MAPK (Erk1/2; p-ERK, 1:1000, 4370, Cell Signaling Technologies) and rabbit polyclonal caspase 3 (1:1000, 8G10, Cell Signaling Technologies). Secondary antibodies were HRP-conjugated donkey anti-rabbit-IgG and sheep anti-mouse-IgG (both from GE Healthcare, Helsinki, Finland). The antibodies were detected with Enhanced Chemiluminescence kit (Thermo Fischer Scientific).

### 2.8. Micro Electrode Array (MEA) Electrophysiology

Spontaneously contracting cardiac aggregates were micro-dissected and plated on 0.1% gelatin coated 6-well micro electrode arrays (MEAs); (MEA1060-Inv-BC, Multichannel Systems, Reutlingen, Germany). Recordings were performed in serum free embryoid body (EB) medium consisting of KO-DMEM, nonessential amino acids (Lonza, Basel, Switzerland), GlutaMAX Supplement (Gibco) and penicillin/streptomycin (Lonza) at 36 ± 1 °C (Temperature controller, TC02, Multichannel Systems, Germany). The MEAs were covered with gas permeable fluorinated ethylene-propylene membranes (ALA MEA-MEM-sheet, ALA Scientific, Farmingdale, NY, USA) and after a 30 min stabilization period, the baseline recordings were performed for at least 20 min. Adrenaline (Sigma-Aldrich) was applied after baseline recording to a final concentration of 100 nM and 1 µM and the effect was recorded at least for 20 min. Output signals were digitized at 10 kHz by the use of a computer equipped with a MC-card data acquisition board (Multi Channel Systems, Reutlingen, Germany). MEA data analysis of the recorded field potential data was analyzed using a custom developed analysis module in Origin 2017 (Microcal OriginTM, Northampton, MA, USA). Beating frequency by beats per minutes (BPM) and field potential duration (FPD) were extracted from the data (Figure S6). The Bazett’s formula was used to calculate the beat rate corrected FPD (cFPD). Beat rate variation was determined by measuring the variation in randomly selected ≥30 consecutive inter-beat intervals calculated by BRV = ∑│D n + 1 − D n│/[30 × √2] [31] using a custom built algorithm.

### 2.9. Hypoxic Stress Induction on MEA

A five-day follow-up experiment was carried out by modifying the oxygen concentration at regular intervals on the 1-well MEA using a custom built hypoxia chamber [32]. A non-humidified and filtered, hypoxia gas mixture (1% O_2_, 5% CO_2_, 94% N_2_) or normoxia gas mix (19% O_2_, 5% CO_2_, 76% N_2_) at a flow rate of 5 mL/min (350 mbar pressure) was supplied to the aggregates placed in serum-free EB formation medium. The measurements were done for five continuous days. On the first day, the concentration of oxygen in the gas mix was kept at 19% and on day 2, 3 and 4 the aggregates were subjected to a cycle of hypoxia (1% oxygen) for 3 h and overnight normoxia (19% oxygen).

### 2.10. Calcium Imaging

Intracellular calcium handling was studied using single wavelength fluorescent Ca^2+^ dye Fluo-4 AM (acetoxymethyl ester); (Thermo Fisher Scientific) diluted in anhydrous DMSO (Thermo Fisher Scientific) and imaged with Axio Observer 1A inverted fluorescence microscope (Carl Zeiss CMP GmBH, Gottingen, Germany) equipped with an ANDOR iXON3 camera (Andor technology, Belfast, Ireland). Cardiomyocyte aggregates were dissociated into single CMs onto 0.1% gelatin coated 12 mm glass coverslips. Four to seven days post-dissociation, CMs were loaded with 4 µM Fluo-4AM in a HEPES based medium for 30–45 min before imaging. The coverslip was transferred to RC-25 perfusion chamber (Warner Instruments Inc., Hamden, CT, USA) perfused with extracellular solution and preheated by an SH-27B inline heater (Warner Instruments Ltd.) to 36 ± 1 °C. The extracellular solution consisted of (in mmol/L): 137 NaCl, 5 KCl, 0.44 KH_2_PO_4_, 20 HEPES, 4.2 NaHCO_3_, 5 D-glucose, 2 CaCl_2_, 1.2 MgCl_2_ and 1 Na-pyruvate (pH 7.4) and osmolarity at 310 mOsm/KG. The imaging was done using a 20× objective and FITC filter with the excitation-emission wavelength of 494/506 nm. The imaging was done for 30–40 seconds captured at a 50 FPS frame rate. Imaging software Zen 2.3 software (Zeiss, Jena, Germany) was used and the files were acquired in .CZT file format. Mean intensity of the calcium transients was analyzed by drawing a region of interest (ROI) over the whole cells. The background noise was subtracted before further processing. The Ca^2+^ levels are presented as ratiometric values of ΔF/F_0_. Recording was done before and 3–5 min after administration of 10 nmol/L adrenaline (Sigma-Aldrich). The Ca^2+^ transients were analyzed with Clampfit version 9.2 (Molecular devices, San Jose, CA, USA).

### 2.11. Teratoma Assay

Approximately 200,000 morphologically intact iPSCs were intratesticularly injected into male NODSKIDgamma (NSG) mice. The tumours were removed two months after injection, fixed in 4% paraformaldehyde and embedded in paraffin. Histological sections were cut at 4 µm and stained with hematoxylin and eosin. Animal care and experiments were approved by the National Animal Experiment Board in Finland (ESAVI/9978/04.10.07/2014).

## 3. Results

### 3.1. Generation and Characterization of hiPSC Lines

Biopsies from two healthy controls and two DCM patients (DCM1 and DCM2) with a heterozygous p.S143P mutation in *LMNA* were used to generate hiPSC clones. The basic characterization of one control line (Control1) has been previously published [22]. Similarly, the other three lines showed typical characteristics of hiPSC morphology without detectable differences in the expression of pluripotency markers (Figure S1A,B). A normal karyotype was confirmed in all the lines (data now shown). The expression of endogenous stem cell markers (REX1, SOX2, NANOG and c-Myc) and the absence of exogenous transcripts were further confirmed with qRT-PCR in all the lines (Figure S1C,D). In the teratoma assay, tissues derived from each of the embryonic germ layers (endoderm, mesoderm and ectoderm) were observed (Figure S1E). Similarly, embryoid body (EB) differentiation confirmed the expression of three germ layer markers in RT-PCR (Figure S2). The expression of p.S143P mutant lamin A/C mRNA was detected in DCM-CMs but not in controls (Figure S3). The presence of the p.S143P (TCC to CCC) mutation in the *LMNA* was also confirmed in the hiPSC lines derived from DCM patient1 and 2 by DNA sequencing (Figure S4).

### 3.2. Characterization of Cardiac Differentiation and Lamina Structure

**Figure 1:**
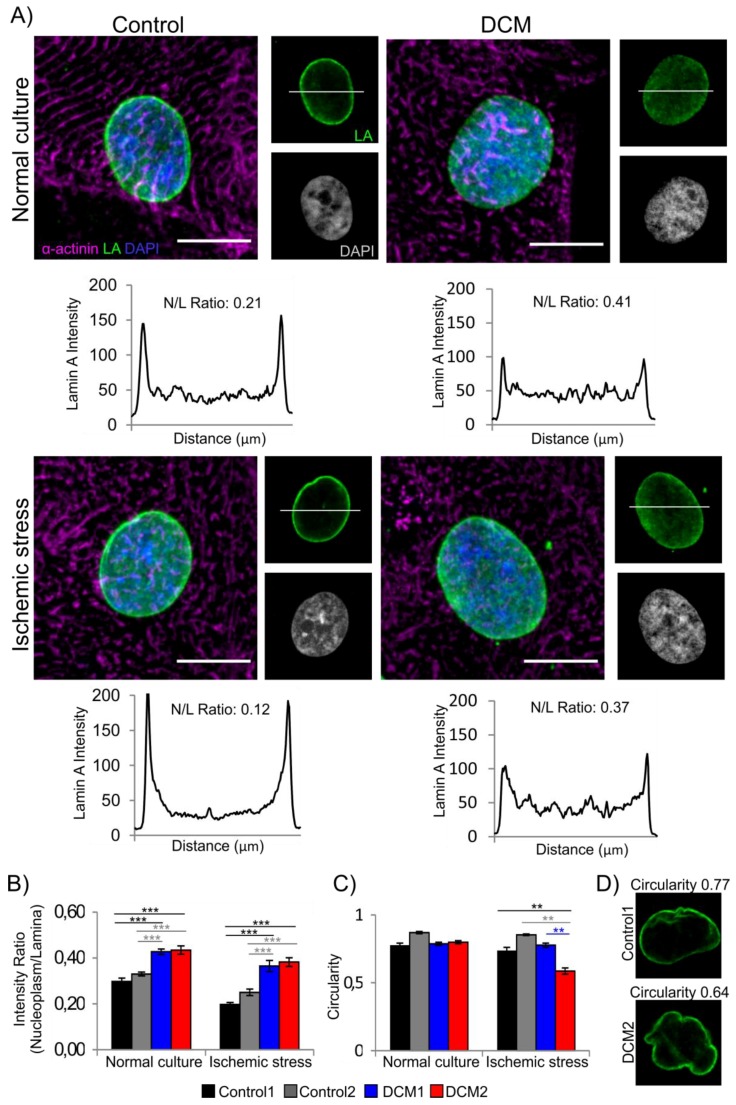
Characterization of lamina structure in human induced pluripotent stem cell (hiPSC) derived cardiomyocytes. (**A**) Dissociated control and dilated cardiomyopathy (DCM) hiPSC-cardiomyocytes (CMs) were cultured either in normal culture conditions or exposed to ischemic stress for 3 h, fixed and stained for lamin A (LA, green), α-actinin (magenta) and DNA (DAPI; blue). Representative maximum projections of Z-stack sections (merged) and single mid-plane confocal sections (LA, DAPI) from control1 and DCM2 CMs are shown. Scale bar 10 µm. Fluorescent intensity values are illustrated below the image with nucleoplasm/lamina (N/L) ratio numbers. (**B**) Fluorescence intensities at the lamina region and in the nucleoplasm were determined from mid-plane confocal sections of 20–30 randomly selected cells and the average ratios of the signals (nucleoplasm/lamina) were plotted. (***) *p* < 0.001. (**C**) Quantification of nuclear circularity. (**) *p* < 0.01 and (***) *p* < 0.001. (**D**) Example images of different circularity values corresponding to the calculated average values (Perfect circle = 1.0).

All hiPSC clones were further differentiated towards cardiac phenotype and analyzed under normal and hypoxic culture conditions. On an average, 80–98% of cells stained positively with cardiac specific structural protein, α-actinin, except control2 where only 55% of the cells stained positive for cardiac marker (data not shown). Based on confocal microscopy analysis, DCM-CMs had more nucleoplasmic lamin A both under normal culture conditions and after ischemic stress when compared to controls (*p* < 0.001 for all, *n* = 20–30, Figure 1A,B). There were no significant differences in nuclear size or shape between cell lines under normal culture conditions (Figure 1C and Figure S5). However, DCM2-CMs showed significantly more deformed nuclei after ischemic stress, as assessed by decreased circularity (*p* < 0.001, *n* = 20–30, Figure 1C,D). DCM1-CMs, on the other hand, retained their circularity after hypoxia suggesting that they may be less sensitive to hypoxic conditions.

### 3.3. DCM-CMs Exhibit Increased Arrhythmias on MEA

**Figure 2:**
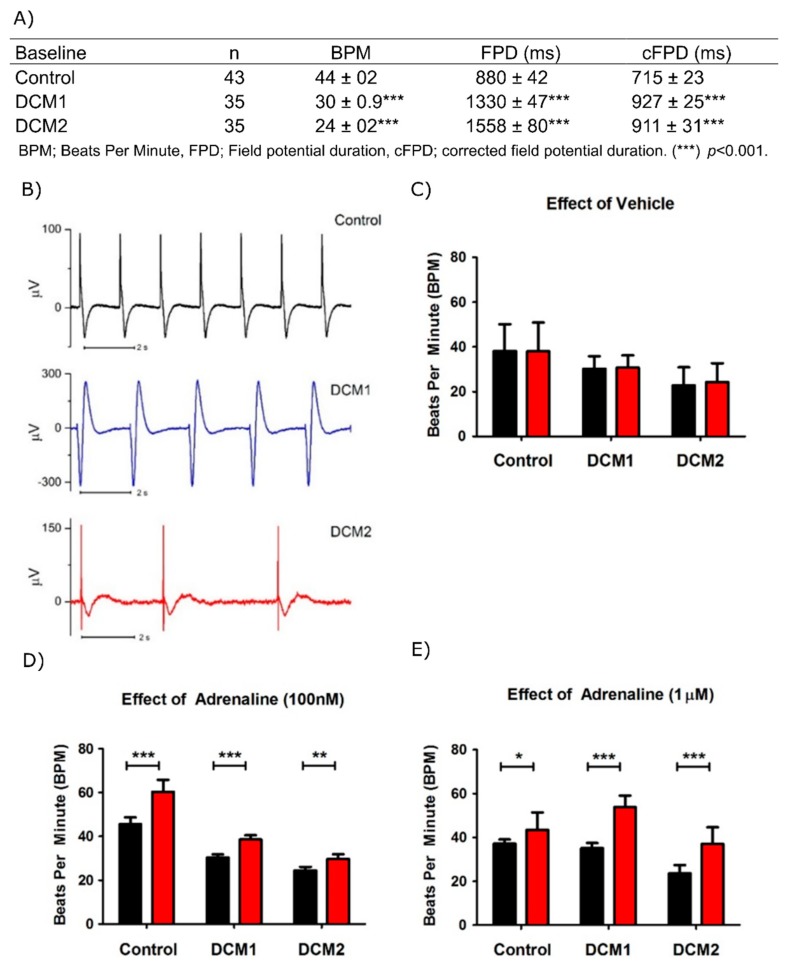
Functional characterization of hiPSC derived cardiomyocytes. (**A**) Table shows baseline electrophysiological characteristics by microelectrode array (MEA) from spontaneously beating cardiomyocyte clusters. (**B**) Representative field potential traces from control, DCM1 and DCM2 cardiomyocyte clusters at baseline. (**C**–**E**) The bar charts show chronotropic responses of control, DCM1 and DCM2 hiPSC-CM clusters to distilled water used as vehicle (*n* = 23, 16, 16 respectively); 100 nM adrenaline (*n* = 24, 26, 18 respectively) and 1µM adrenaline (*n* = 18, 16, 13 respectively) with black and red bars showing the beating frequency at baseline and under vehicle/adrenaline respectively. Combined results from Control1 and Control2 are shown. Data is presented as mean ± s.e.m., (*) *p* < 0.05, (**) *p* < 0.01 and (***) *p* < 0.001.

Cardiac functional phenotype was confirmed by generation of field potentials at multi-cellular level on the micro electrode array (MEA). Based on the baseline electrophysiological characteristics, the DCM-CM aggregates showed a significantly reduced beating rate and a significantly increased field potential duration compared to the aggregates from controls (Figure 2A,B). To analyze the presence of functional β-adrenergic receptors, adrenaline was applied on the beating aggregates. β-adrenergic stimulation with adrenaline showed a positive chronotropic response in all CM aggregates, whereas the vehicle (distilled water) had no effect on the beating rate (Figure 2C–E).

**Figure 3:**
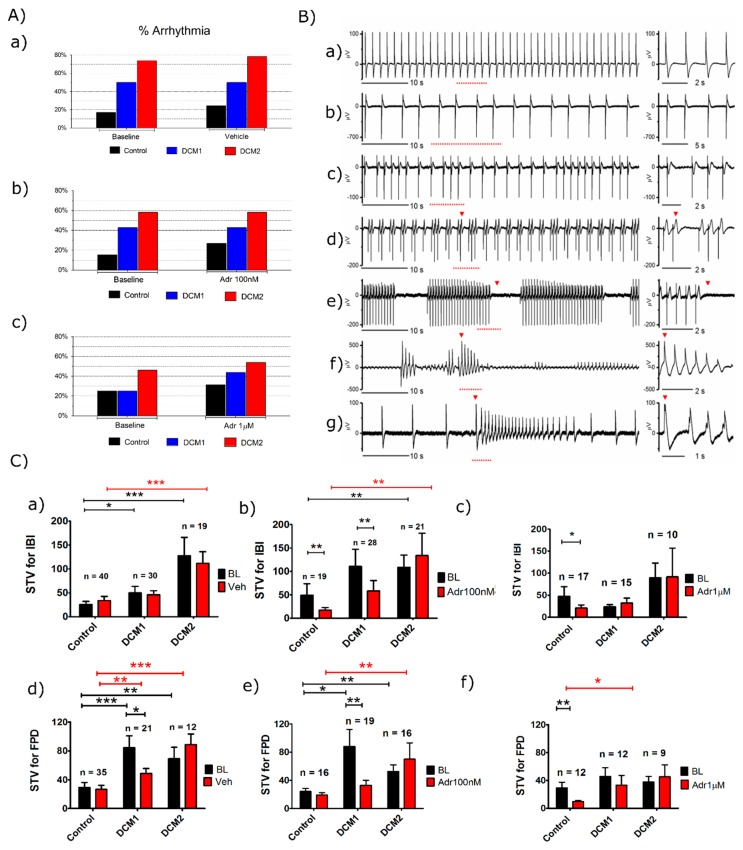
Arrhythmia occurrence in hiPSC-CM beating aggregates on MEA. (**A**) Percentage of arrhythmic aggregates compared to baseline after the addition of a) vehicle (Control, DCM1 and DCM2, *n* = 41, 26 and 16 respectively), b) 100nM adrenaline (Control, DCM1 and DCM2, *n* = 32, 28 and 16 respectively) and c) 1µM adrenaline (Control, DCM1 and DCM2, *n* = 23, 24 and 13 respectively). (**B**) Representative MEA extracellular recordings from DCM hiPSC-CMs after application of adrenaline. A 60 second MEA trace is shown on left and a magnified view of the corresponding signal (red dotted lines) on the right. The representative traces showing a normal beating rhythm (a), alternations (b), dysrhythmia (c), premature beat (d) and ventricular-tachycardia like arrhythmia (e–g). The red arrows indicate arrhythmia other than alternations or dysrhythmia. (**C**) An irregular beating pattern observed in DCM1 and DCM2 aggregates was quantified for ≥30 consecutive beats by the formula for short term variation (STV) of inter-beat interval (a–c) and STV of field potential duration (d–f). BL signifies baseline, veh is vehicle and Adr is adrenaline. Data is presented as mean ± s.e.m. ANOVA non-parametric tests and a paired *t*-test were used in statistical analysis; (*) *p* < 0.05, (**) *p* < 0.01 and (***) *p* < 0.001. Combined results from Control1 and Control2 are shown.

The field potential recordings on MEA revealed that aggregates from DCM-CMs showed increased arrhythmicity at baseline. The application of adrenaline gave a more pronounced arrhythmic phenotype with elevated beating irregularity and arrhythmic beats in the DCM-CM aggregates compared to controls (Figure 3A). Apart from a regular positive chronotropic response (Figure 3B(a)), different types of arrhythmias were observed, including alternations and irregularity (Figure 3B(b,c)), more severe arrhythmias similar to the occurrence of a premature beat (Figure 3B(d,e)) and ventricular tachycardia like arrhythmias (Figure 3B(f,g)). The less severe arrhythmias like alternations and irregularity were more commonly observed in the controls while more severe arrhythmias were typical for DCM-CM aggregates.

At baseline the variation in the beating rhythmicity, calculated by short term variation (STV), was found to be significantly elevated for the inter-beat interval (IBI) and field potential duration (FPD) in both DCM1-CM and DCM2-CM aggregates compared to controls (Figure 3C(a,d)). Application of 100 nM adrenaline led to a decrease in the STV for IBI and STV for FPD in control and DCM1, but had no significant effect on DCM2 (Figure 3C(b,e)). Similarly, a higher 1µM concentration of adrenaline significantly decreased the STV for IBI and STV for FPD in the control CM aggregates while little or no effect was seen on DCM1 and DCM2 (Figure 3C(c,f)). These results from field potential recordings suggest that DCM-CMs have increased irregularity at baseline and they are more prone to arrhythmias on adrenaline application, showing a similarity with ECG findings from patients with DCM due to *LMNA* mutation.

### 3.4. Increased Sarcomere Disorganization after Ischemic Stress in DCM-CMs

**Figure 4:**
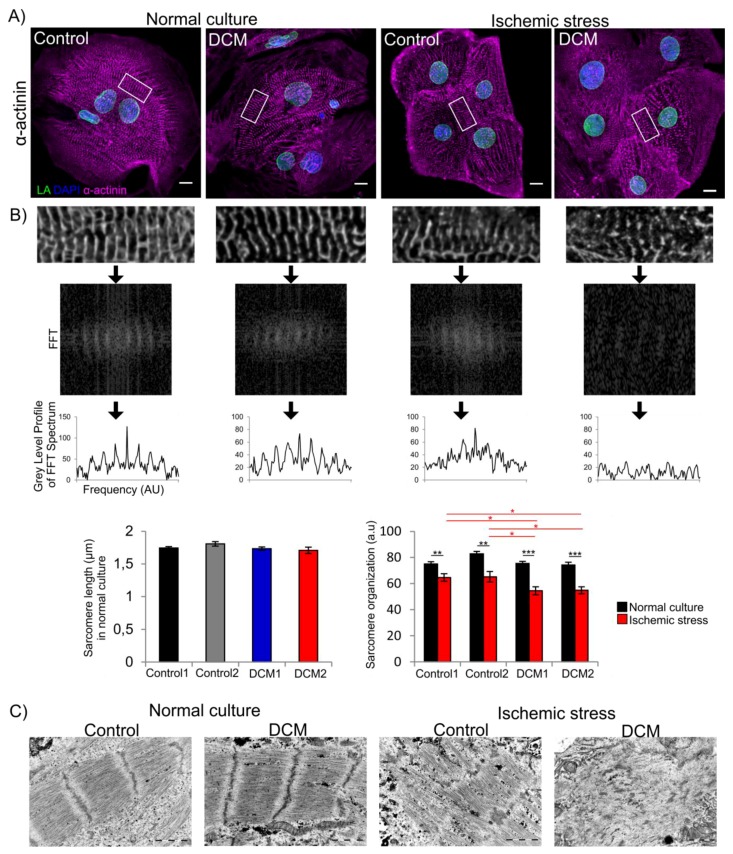
Confocal microscopy analysis of hiPSC-CM sarcomere structure under normal and hypoxic conditions. (**A**) Control and DCM CMs were cultured under normal culture conditions or exposed to ischemic stress, fixed and stained for α-actinin, LA and DAPI. Representative maximum projections of Z-stack sections from control1 and DCM2 are shown. Scale bar: 10 µm. (**B**) TT power analyses of the sarcomere length and organization were carried out with TTorg plugin in ImageJ. TTorg workflow of the example images: Magnification of an original image, 2D fast Fourier transformation (FFT) spectrum of the image, grey level profile of the FFT spectrum and analysis results (*n* = 20, AU = Arbitrary Units). Data is expressed as mean ± s.e.m.,* *p* < 0.05. (**C**) Transmission electron microscopy images of control1 and DCM2 CMs under normal culture conditions and ischemic stress are shown. Scale bar: 1 µm.

In order to test whether a mutation in lamin A/C affects the structural protein organization, we analyzed the sarcomere length and organization in control and DCM-CMs by confocal microscopy and automated image analysis from α-actinin stained cells (Figure 4A). Under normal culture conditions, there were no significant differences in either sarcomere length or organization between the cell lines (Figure 4A,B). The stability of sarcomere structure was further tested by exposure to 3 h of ischemic stress. Interestingly, we observed a dramatic sarcomere disorganization (scattering and loss of repeatability formed by Z-lines) in the majority of DCM-CMs while the changes were less pronounced in control CMs (Figure 4A). These differences were also found significant in image analysis carried out on randomly selected cells (*p* < 0.05; *n* = 20 in each group; Figure 4B). Similarly, transmission electron microscopy analysis showed no difference between control and DCM-CM sarcomere organization at baseline. However, after ischemic stress, the sarcomeres were notably more scattered in DCM-CMs relative to controls (Figure 4C).

### 3.5. DCM-CMs Show Increased Cellular Stress

**Figure 5:**
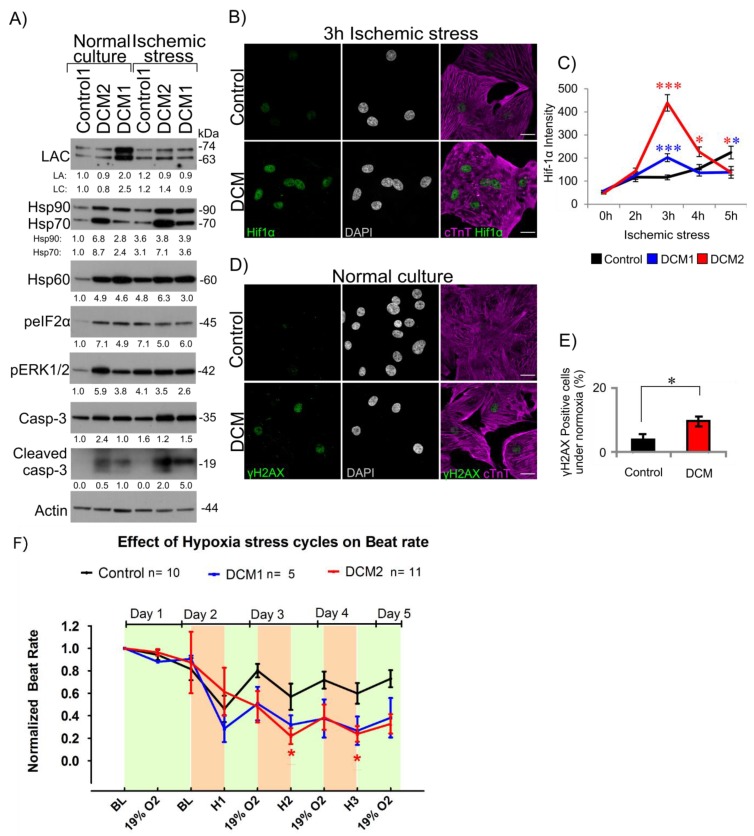
DCM hiPSC-CMs show increased elevated cellular stress. (**A**) Western blot analysis of lamin A/C, phospho-eIF2α (peIF2α), Hsp90, Hsp70, Hsp60, phospho-ERK1/2 (pERK1/2) and cleaved caspase-3 under normal culture conditions and after exposure to ischemic stress for 3 h. Actin was used as a loading control. The average numerical values of signal intensities relative to the loading control (actin) are shown below each blot. *n* = 2 individual experiments. Control2 hiPSC-CMs were not qualified for analysis due to lower differentiation efficiency compared to other lines. (**B**) Confocal microscopy analysis of Hif-1α intensity. Control1, DCM1 and DCM2 hiPSC-CMs were cultured either in normal culture conditions or exposed to ischemic stress for 2 h, 3 h, 4 h and 5 h, fixed and stained for Hif-1α, cardiac marker cTnT and DNA (DAPI). A 3 h time point is shown. Scale bar 20 µm. (**C**) The fluorescence intensities of Hif-1α were determined from all the confocal sections of >15 randomly selected cells at different time points and the average normalized signals were plotted. (**D**) Control1 and DCM2 hiPSC-CMs were cultured under normal culture conditions, fixed and stained for γH2AX, cTnT and DNA (DAPI). (**E**) γH2AX positive cells from control1 and DCM2 were counted and plotted (*n* = 500). (**F**) Effect of three repeated 3 h cycles of hypoxia (1% O_2_) shown as H1, H2 and H3 and overnight re-oxygenation (19% O_2_) on beat rate of hiPSC-CMs recorded on MEA. Control data presented in F is combined from Control1 and 2. Data is expressed as mean ± s.e.m., (*) *p* < 0.05, (**) *p* < 0.01 and (***) *p* < 0.001.

To investigate the overall cellular stress in DCM aggregates further, the abundance of various stress-related markers was studied by western blotting both under normal culture conditions and after 3 h of ischemic stress (Figure 5A). CMs from both DCM1 and DCM2 exhibited clear upregulation of endoplasmic reticulum (ER) stress marker phosphorylated translation initiation factor 2α (peIF2α) and heat shock proteins 90, 70 and 60 under normal culture conditions. After 3 h of ischemic stress, peIF2α and Hsp90, 70 and 60 levels increased in controls and were highly similar to those observed in mutant cells. In addition, phosphorylated extracellular signal-regulated protein kinases 1 and 2 (ERK1/2) were significantly upregulated in both DCM-CM under normal culture conditions indicating activation of mitogen-activated protein kinase (MAPK) and a similarity with previously reported *Lmna*^H222P/H22P^ DCM mouse model [16].

Confocal microscopy analysis revealed that the hypoxia inducible factor 1α (Hif-1α) was significantly more upregulated in DCM than in control cells after 3 h of ischemic stress (*p* < 0,001; *n* > 15, Figure 5B). More specifically, Hif-1α level increased exponentially after 2 h of hypoxia, reached its peak value at 3 h and decreased substantially thereafter while in control cells only moderate accumulation was observed after 4 h hypoxia (*p* < 0,05; *n* > 20, Figure 5C).

Finally, we wanted to test whether increased cellular stress would have any effect on DNA damage and cell viability. The number of cells positive for phosphorylated histone H2AX (γH2AX), a marker for double-strand DNA breaks, was significantly increased in DCM cells compared to control under normal culture condition (*p* < 0.05; *n* = 500; Figure 5D,E). However, no significant difference was found after ischemic stress as virtually all the DCM and control cells stained positively (data not shown).

Based on the increased levels of cleaved (active) caspase-3, there also appeared to be the induction of apoptotic cell death among DCM1 and DCM2 CMs already under normal culture conditions and more distinctly after ischemic stress while such cleavage was not detected in control CMs (Figure 5A). In summary, these findings suggest that DCM-CMs exhibit elevated baseline cellular stress that may sensitize to programed cell death upon additional physiological stress.

### 3.6. Hypoxia Reduces Beat Rate of Cardiac Aggregates

To test whether hypoxia induced stress response has any effect on the function of hiPSC-CMs, cardiac aggregates on MEA were exposed to repeated cycles of hypoxia (1%, 3 h) on three consecutive days, followed by overnight recovery in normoxia. Hypoxia stress induction assay by a custom built hypoxia chamber on the MEA was used for this recording (Figure S7). The main observation was that hypoxia reduced the beating rate in all the hiPSC-CM aggregates (Figure 5F). However, the second and third hypoxia cycle had an exaggerated effect on DCM1 and DCM2 aggregates, with DCM2 having significantly reduced beat rate at second and third hypoxia cycle (Figure 5F). Interestingly, overnight recovery in normoxia almost completely restored the functionality in controls but little or no effect was observed on DCM1 and DCM2 aggregates (*n* = 10 control, *n* = 5 DCM1, *n* = 11 DCM2). After three hypoxia–normoxia cycles on day 5, the beating rate in controls was about 80% of the original beating rate, while it was between 30–45% in DCM aggregates indicating an increased sensitivity to hypoxia induced stress (Figure 5F).

### 3.7. DCM-CMs Show Impaired Calcium Dynamics

**Figure 6:**
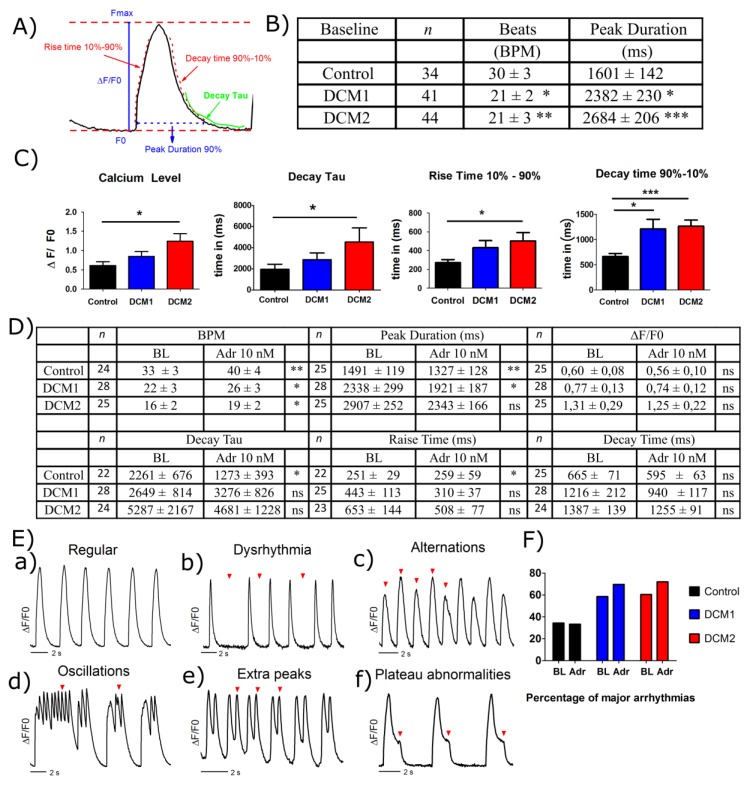
Analysis of intracellular calcium dynamics by calcium (Ca2^+^) imaging in hiPSC-CMs. (**A**) Measurement of Ca^2+^ transient parameters ∆F/F0, peak duration, rise time, decay time and decay tau for the calcium imaging data. (**B**) Ca^2+^ transient characteristics recorded from hiPSC-CMs at baseline. (**C**) Calcium imaging parameters for control, DCM1 and DCM2 CMs at baseline are shown as ∆F/F0 (*n* = 34, 40 and 42 respectively), decay tau (*n* = 31, 41 and 41 respectively), rise time (*n* = 32, 39 and 40 respectively) and decay time (*n* = 31, 34 and 35 respectively). (**D**) Calcium imaging parameters at baseline (BL) and in the presence of 10 nM adrenaline. (**E**) Representative regular (a) and arrhythmic Ca^2+^ transients (b–f) detected under baseline or under 10 nM adrenaline in control, DCM1 and DCM2 CMs. Dysrhythmia (b) and alternations (c) were categorized as minor arrhythmias and oscillations (d), extra peaks (e) and plateau abnormalities (f) as major arrhythmias. The red arrows represent the Ca^2+^ abnormalities. (**F**) Frequency of major arrhythmias in control, DCM1 and DCM2 single dissociated hiPSC-CMs at baseline (BL) and under 10 nM adrenaline (Adr). Data is presented as mean ± s.e.m and ANOVA or non-parametric pair test was used for statistical analysis. (*) *p* < 0.05, (**) *p* < 0.01 and (***) *p* < 0.001. Control data presented here is combined from Control1 and Control2.

To understand the functionality and calcium handling characteristics, single dissociated hiPSC-CMs were studied using Ca^2+^ imaging. A representative calcium transient with measured parameters is shown in Figure 6A. The basal calcium transient characteristics of DCM-CMs showed a significantly reduced beating rate and increased calcium transient peak duration when compared to control (Figure 6B). These results are consistent with results from cardiac aggregates on MEA displaying decreased beating rate (Figure 2A). At baseline the Ca^2+^ levels calculated by ∆F/F_0_, calcium transient decay tau, rise time and decay time were also elevated in DCM-CMs compared to controls (Figure 6C).

To test the effect of adrenergic stress, the calcium transient characteristics were determined under 10 nM adrenaline. Adrenaline increased the mean beat rate (BPM) significantly in all the lines and reduced the mean calcium transient peak duration in control and DCM1 significantly while only a similar trend was seen in DCM2 (Figure 6D). The calcium levels by ∆F/F_0_ and decay time also reduced in all cell lines, but this did not reach statistical significance. The decay tau decreased and rise time increased significantly in controls but neither of these were found significantly altered in DCM-CMs (Figure 6D).

Apart from the regular calcium transients (Figure 6E(a)), different arrhythmias were observed on calcium transients comprising of irregular beating and alternations (Figure 6E(b,c)) as well as more severe arrhythmias including oscillation, extra peaks and plateau abnormalities (Figure 6E(d–f)). Increased frequency of major arrhythmias was observed in DCM-CMs already at baseline (Figure 6F) and β-adrenergic stimulation seemed to increase their occurrence. However, no clear effect was observed in control CMs upon adrenaline treatment (Figure 6F).

## 4. Discussion

In this work, we have generated and characterized a novel hiPSC-CM model carrying the p.S143P *LMNA* mutation.

Structural analysis showed that the sarcomeres were highly organized in DCM-CMs and were indistinguishable from control-CMs. However, after ischemic stress α-actinin staining demonstrated significantly more disrupted sarcomere organization in DCM-CMs (Figure 4). Previously, a similarly disrupted sarcomere organization was observed in CMs carrying the *LMNA* p.R190W mutation but these differences were detectable already under normal culture conditions [33]. These phenotypic cellular differences between lamin mutations may be due to differences in pathogenicity and assembly properties of mutant lamin protein as they may have different effects e.g., on the organization of LINC (linker of nucleoskeleton and cytoskeleton) complexes. 

Similar to our earlier observations on p.S143P mutant primary fibroblast [25], DCM-CMs had significantly more nucleoplasmic lamin A/C relative to controls (Figure 1A,B). Since p.S143P lamin A/C is incapable of forming regular lamin filaments in vitro [25] and the wild type and mutant allele were transcribed in an equal manner (Figure S3), it is plausible that the excess nucleoplasmic lamin pool is formed by the mutant protein. Vice versa, such mislocalization shall lead to reduced amounts of lamin A/C at the lamina region, as pointed out by our analysis (Figure 1B). Furthermore, this may influence lamina stability and lead to deformed nuclei under stressed conditions as observed in the circularity analysis of DCM2-CMs (Figure 1C,D). In summary, these results indicate that while control CMs are capable of adapting to stress by keeping their lamina intact, the *LMNA*-mutant DCM-CMs are more sensitive due to insufficient amounts of lamin A/C (i.e., haploinsufficiency) and potentially fragile lamina. This may eventually cause DNA damage and cell death especially in tissues that are exposed to constant stress, such as in the cardiac muscle. The latter is also supported by increased γH2AX staining (Figure 5D,E) and caspase-3 activity (Figure 5A) in DCM-CMs upon hypoxia.

Immunoblotting revealed significant upregulation of several stress related signaling proteins such as Hsp90, Hsp70 and Hsp60 in DCM-CMs under normal culture conditions (Figure 5A). Hsp70 and Hsp90 protein family members are essential chaperones in cardiomyocytes and they have a critical role in maintaining cardiac function during stress. Hsp70 (Hsp72 isoform) is only expressed under stress to support correct protein folding and to deliver proteins for proteosomal degradation [34]. Studies on Hsp70 and heart failure have shown conflicting results. Some studies have reported increased Hsp70 mRNA or protein levels among DCM patients [35,36] while this has not been validated by others [37,38]. Nevertheless, accumulating data suggests that Hsp70 plays an essential role in many signaling pathways related to myocardial ischemia/reperfusion (I/R) injury, oxidative stress, Ca^2+^ overload and apoptosis. Chaperone Hsp90 is involved in many cellular processes including protein folding, protein degradation and signal transduction [39] and may also have a cardioprotective role in I/R injury [34,40,41]. Similarly, Hsp60 is upregulated among DCM patients [38,42] but may have both pro- and anti-apoptotic roles depending on cell type [39]. Therefore, constant upregulation of Hsp family members may have both beneficial and deleterious effects on lamin mutant CMs.

Our previous study showed that endogenous and ectopic expression of p.S143P lamin A leads to hyperphosphorylation of eIF2α in fibroblasts [25]. Similarly, elevated levels of peIF2α were detected in DCM-CMs but not in control CMs under normal culture conditions (Figure 5A). Typically eIF2α is phosphorylated upon activation of unfolded protein response (UPR) or ER stress, caused by an accumulation of unfolded or misfolded proteins in the ER. The UPR and ER stress have been linked with the pathophysiology of heart failure and this may be one of the cellular mechanisms that trigger and promote cardiomyocyte apoptosis in failing human hearts [43]. Meanwhile, Hsp70 and Hsp90 bind and stabilize ER-stress sensor proteins, such as PERK and IRE1α, to enhance adaptation to ER-stress and inhibit apoptosis [44,45]. It is currently unclear whether expression of mutant lamin A/C directly or indirectly triggers UPR (e.g., due to misfolding) and whether targeting these complex signaling mechanism would improve CM functionality. However, in patient fibroblasts treatment with chaperone 4-phenylbutyric acid inhibited aggregation of mutant lamin and improved viability [25], which supports testing similar drugs on DCM-CMs in the future. We also observed significant upregulation of pERK1/2 in both DCM-CM lines under normal culture conditions. This result correlates with previous data from the *Lmna*^H222P/H22P^ mouse model [16] and further supports the involvement of MAPK pathway in the pathobiology of *LMNA*-related DCM.

Reduction of oxygen forces the cardiomyocytes to switch from aerobic to anaerobic respiration pathways, leading to an increase in glycolytic substrates like lactate [46,47] while perturbation on ATP generation affects cardiac contractility [48]. In our study, repeated hypoxia and normoxia cycles mimicking I/R injury reduced the beating in all aggregates but had more severe effects on DCM-CMs with only 30–45% recovery of their original beat rate (compared to 80% in controls, Figure 5F). Increasingly compromised sarcomere structure due to hypoxic insults, as well as increased DNA damage and constant upregulation of stress proteins such as Hif1α and HSP70 [46] thus could explain the low recovery of the beat rate in DCM-aggregates and the initiation of arrhythmias on multicellular and single CM level. This experiment further demonstrates the feasibility of hiPSC-CMs for cardiac disease and I/R injury modeling to advance our understanding on cardiac pathophysiology.

Ca^2+^ imaging studies revealed elevated cytosolic calcium levels and increased decay tau, rise time and decay time in DCM-CMs compared to controls. Similarly Nikolova et al. reported significantly increased time to 50% relaxation of the Ca^2+^ transient in *LMNA*^+/−^ and *LMNA*^−/−^ mice’ CMs [17]. However, no difference in baseline Ca^2+^ levels and rise time were observed whereas we found these to be increased in DCM-CMs. Our results are also in line with Ca^2+^ transient data from failing human hearts, where increased time of calcium decay, slow rates of recovery and increased intracellular Ca^2+^ levels have been reported [49]. The rise time of the calcium transient is due to released calcium from the intracellular calcium stores through RYR2 channels, the decay phase is due to removal of Ca^2+^ via SERCA and Na^+^/Ca^2+^ exchanger (NCX) and any of these pathways could be affected in the DCM-CMs [50]. Since ER is also involved in maintaining intracellular Ca^2+^ homeostasis and development of cardiac conductive system [51] and prolonged ER stress has been linked to many pathological cardiovascular diseases [52], further studies are needed to understand the exact links between these mechanisms in lamin mutant CMs.

MEA recordings revealed a reduced beating rate and increased field potential durations in DCM-CM aggregates compared to controls. Similarly, ventricular CMs from *Lmna*^N195K/N195K^ mutant mice showed significantly reduced beating rates, significantly prolonged APD50 and APD90 and occurrence of EADs (early afterdepolarization) and DADs (delayed afterdepolarization) on stimulation [53]. During β-adrenergic stimulation, increased Ca^2+^ load beyond the sarcoplasmic reticulum threshold may lead to pronged action potentials, varying spontaneous calcium release, increased beat rate variation and arrhythmias [49,54,55]. In our Ca^2+^ imaging studies, β-adrenergic stimulation clearly increased arrhythmias in DCM-CMs but had no significant effect on control-CMs (Figure 6). In analogue, increased premature beats and VT-type arrhythmias were observed in DCM-aggregates on MEA when compared to controls (Figure 3). A similar intracellular calcium overload and increased sensitivity to chronotropic stress by norepinephrine was reported in an earlier iPSC-CM model of familial DCM from a *RBM20* mutation [56]. This is of particular interest as reduced RBM20 levels have also been reported in *Lmna*-mutant cells with DCM-related mutations [57]. In our study, single DCM-CMs were more arrhythmic compared to cell clusters on MEA, most likely due to a lack of cell–cell connections [58]. Our results are also in line with previous studies where β-adrenergic stimulation increased the SR calcium load and caused spontaneous calcium release and arrhythmias in rabbit hearts and in a rabbit heart failure model [59,60].

Atrial fibrillation, AV-block, ventricular arrhythmias and non-sustained ventricular tachycardia are frequent even among asymptomatic *LMNA* mutations carriers [61]. Furthermore, case studies have reported increased and more severe arrhythmias in individual patients following physical exercise [62]. At the cellular level, dysrhythmias were quantified by STVs, which were found to be increased in DCM-CM clusters (Figure 3C). The detailed mechanisms of increase in beating rhythm variability at the cellular level remains incompletely understood, but studies point towards irregularity in spontaneous calcium release, calcium regulation and repolarization time [31,54,63]. A previous study on hESC-CM reported that disturbed intra-cellular Ca^2+^ handling increased variation in the beat rate [64]. Significantly increased beat rate variation has also been reported in animal models and in selected human subjects prior to the onset of major arrhythmias [54]. Actually the SVT has consistently been demonstrated to be a more reliable indicator of arrhythmogenic risk than prolonged ventricular repolarization [31,54]. This could explain the increased arrhythmias in DCM-CM aggregates showing increased field potential duration and increased STV in the current study.

## 5. Conclusions

This is the first hiPSC-CM model for p.S143P *LMNA* mutation, a founder mutation affecting several families in Finland. The mutation-specific DCM-CMs demonstrated bradyarrhythmia and severe arrhythmic beats both at the aggregate and single-cell level. The manifestation of the DCM phenotype was supported by data from MEA, Ca^2+^ imaging, western blot and confocal imaging experiments with increased beat rate variability, abnormal calcium handling, increased sensitivity to stress and elevated expression of stress proteins. The hiPSC-CMs further showed an altered lamina structure and increased disorganization of sarcomere structure upon hypoxic stress. This model helps to understand the molecular pathology of *LMNA*-related DCM and provides a platform for drug testing in the future.

### 5.1. Clinical Significance

Currently there are no specific medications for individuals carrying DCM-related *LMNA* mutations to prevent the onset of the disease or for those already having the clinical phenotype. Better knowledge of the disease pathophysiology would enable to design new treatments in a more focused manner and mutation and disease-specific CMs provide a powerful platform for this aim.

### 5.2. Study Limitations

Although hiPSC-CMs present an advantage over existing animal models and expression systems, hiPSC-CMs present batch wise variations in reprogramming and differentiation. The hiPSC-CM also have an immature phenotype compared to adult human cardiomyocytes. Hence care must be taken while extrapolating to clinical data. Investigating the mechanism of beat rate variation, arrhythmia and hypoxia was beyond the scope of this study. Isogenic controls would be ideal but unfortunately they were not available for the current study.

## Supplementary Materials

The following are available online at https://www.mdpi.com/2073-4409/8/6/594/s1, Figure S1: Characterization of hiPSC lines, Figure S2. Embryoid body differentiation assay for detection of ectoderm, mesoderm and endoderm, Figure S3. Genotyping, Figure S4. DNA sequencing, Figure S5. Nuclear Area, Figure S6. Representative field potential trace, Figure S7. The set-up of custom-made hypoxia chamber.
