# Supplementary material for: Modeling of LMNA-Related Dilated Cardiomyopathy Using Human Induced Pluripotent Stem Cells
Source: Cells. 2019 Jun 15;8(6):594. doi: 10.3390/cells8060594 (PMC6627421; doi:10.3390/cells8060594)
Supplement: Supplementary file 1 [file cells-08-00594-s001.pdf]

Shah, Virtanen et al, Supplementary figures

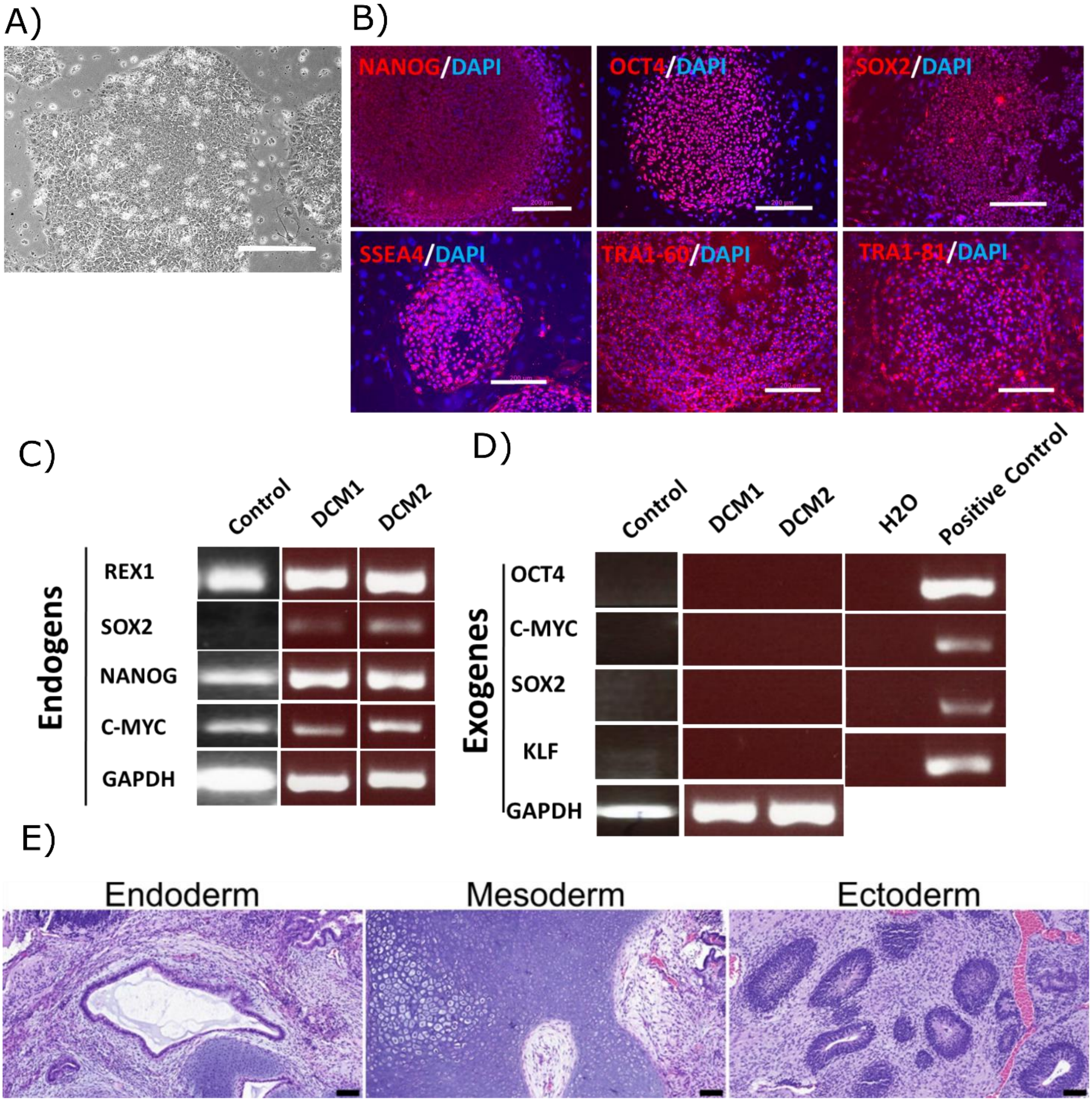

Figure S1: Characterization of hiPSC lines. A) Representative hiPSC colonies from DCM1 under phase contrast microscopy. B) Representative immunofluorescence staining from DCM1 hiPSCs colonies expressing NANOG, OCT4, SOX2, SSEA4, TRA-1-60, and TRA-1-81. Stainings were essentially similar in all the lines. Scale bars: 200 μm. C) Expression of endogens in hiPSC; *REX1* (306 bp), *SOX2* (151 bp), *Nanog* (287 bp), and *c-MYC* (328 bp). *GAPDH* (302 bp) was used as a housekeeping control transcript. D) The virally transfected Sendai exogenes, *exo-OCT4* (483 bp), *exo-c-MYC* (532 bp), exo-*SOX2* (451 bp) and *exo-KLF4* (410 bp), were silenced in the hiPSCs 1 week after transfection GAPDH was used as a control. E) Teratoma assay. hiPSCs were injected intratesticularly into male NODSKIDgamma (NSG) mice and let to form tumours for 2 months. Histological analysis shows teratomas formed by all three germ layers (glandular structures from endoderm, cartilage from mesoderm and neural rosettes from ectoderm). Only teratoma formed by DCM2 hiPSCs is shown as an example. Scale bars 100 μm.

Figure S2. Embryoid body (EB) assay for detection of ectoderm, mesoderm and endoderm. Full characterization of Control1 including EB assay was carried out in a previous manuscript from the same group (Lahti, Kujala et al. 2011, Ahola, Kiviaho et al. 2014).

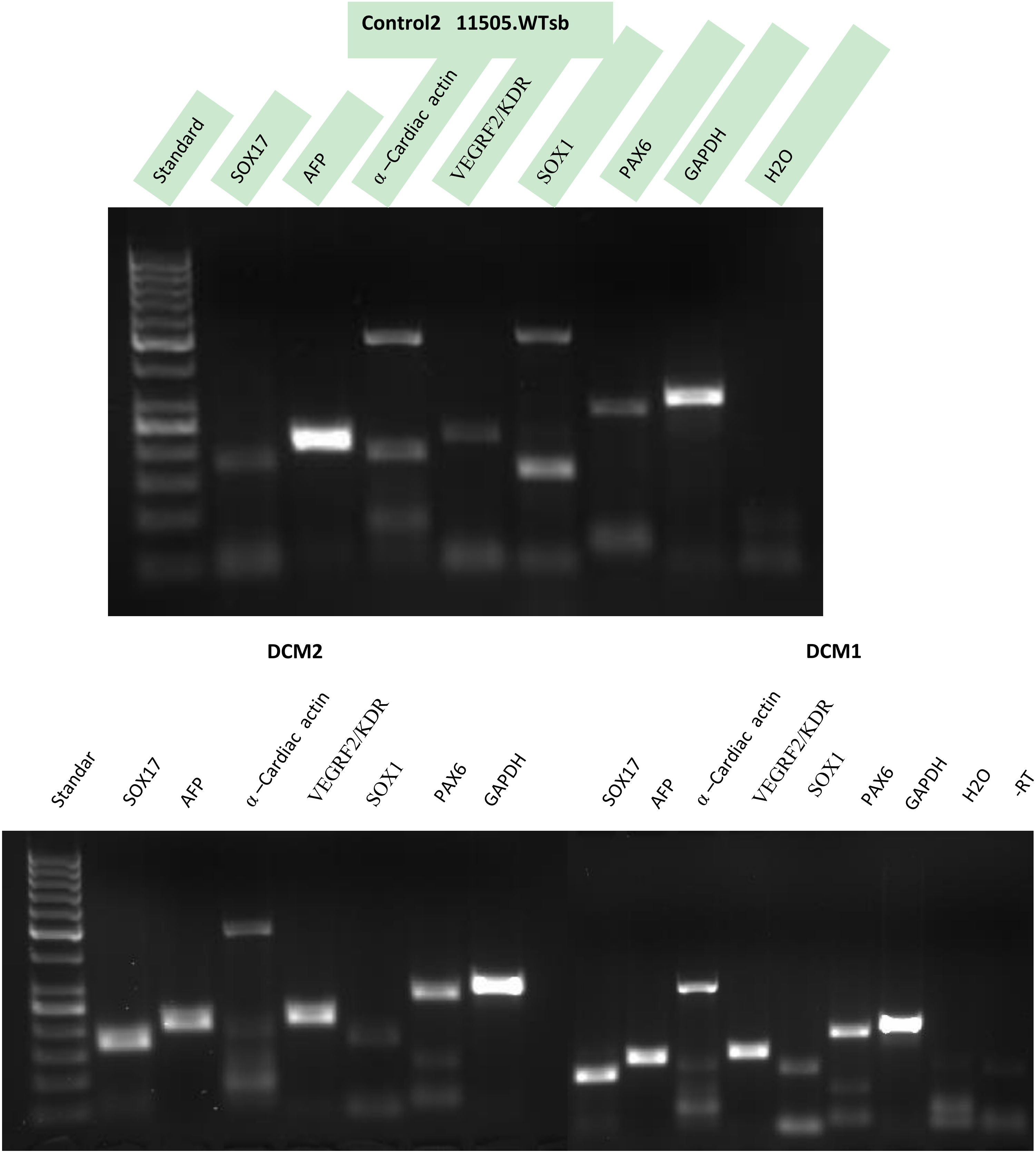

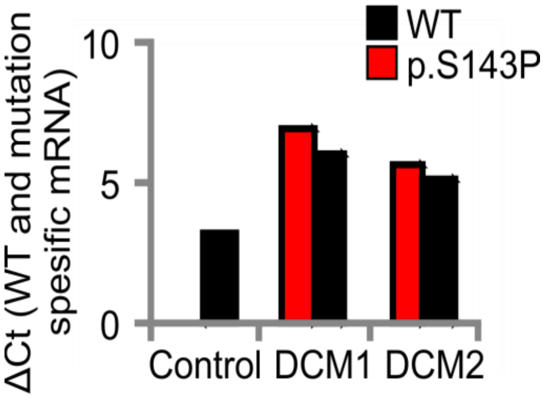

Figure S3. **Genotyping of hiPSC-CM.** The presence of the heterozygous p.S143P *LMNA* mutation was detected with qRT-PCR in mature cardiomyocytes from DCM1 and DCM2 lines but not in controls.

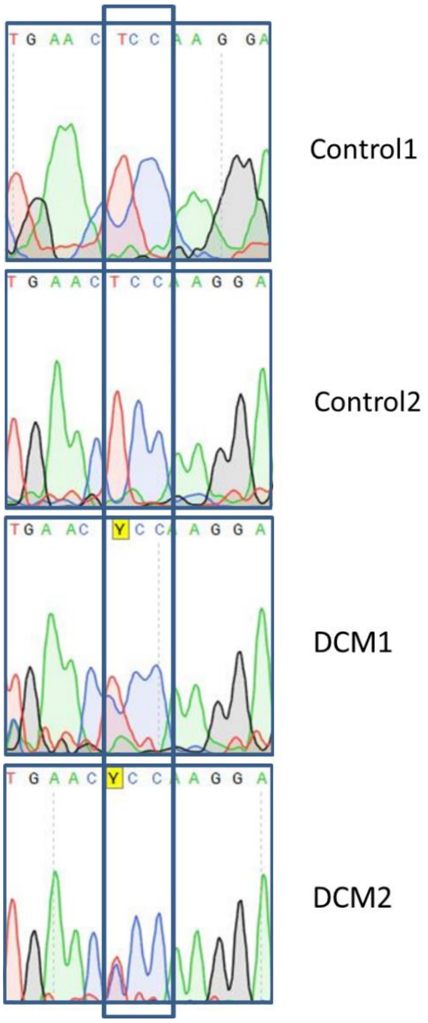

Figure S4. DNA sequencing. The presence of the p.S143P (TCC to CCC) mutation in the *LMNA* was confirmed in the hiPSC lines derived from DCM patient1 and 2 by DNA sequencing. The Y in the yellow box is C and T.

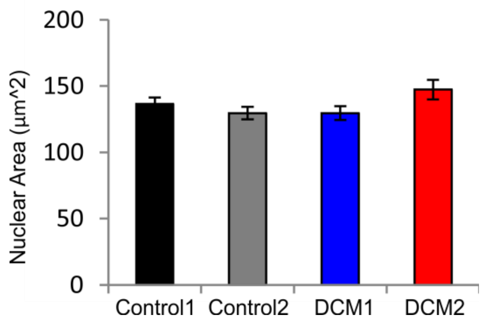

Figure S5. Nuclear Area during normal culture condition (μm ^{2}) N=40-50.

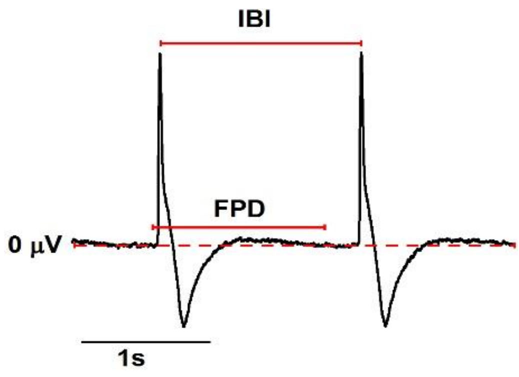

Figure S6. Representative field potential trace. The start and end points of inter-beat interval (IBI) and field potential duration (FPD) are indicated. FPD was calculated from the start of the depolarization wave until the repolarization wave reaching the base.

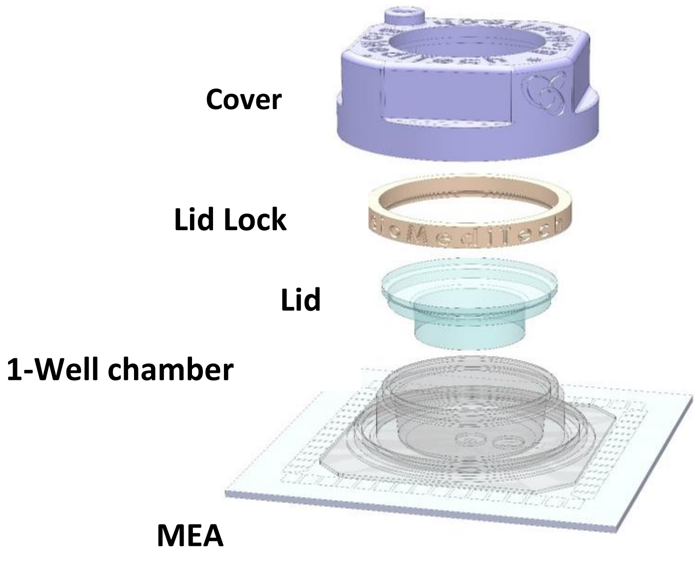

Figure S7. **The set-up of custom-made hypoxia chamber**. Setup to facilitate simultaneous regulation of oxygen at 19% or 1% and recording of field potentials on MEA.
